# Supplementary material for: PANDAA intentionally violates conventional qPCR design to enable durable, mismatch-agnostic detection of highly polymorphic pathogens
Source: Commun Biol. 2021 Feb 18;4:227. doi: 10.1038/s42003-021-01751-9 (PMC7892852; doi:10.1038/s42003-021-01751-9)
Supplement: Supplementary file 2 — Supplementary Information [file 42003_2021_1751_MOESM2_ESM.pdf]

Supplementary Information for

PANDAA intentionally violates conventional qPCR design to enable durable, mismatch-agnostic detection of highly polymorphic pathogens.

Iain J. MacLeod [1,2*], Christopher F. Rowley [1,2,3], M. Essex [1, 2]

1. Department of Immunology and Infectious Diseases, Harvard T.H. Chan School of Public Health, Boston, MA, USA
2. Botswana Harvard AIDS Institute Partnership, Gaborone, Botswana
3. Division of Infectious Diseases, Beth Israel Deaconess Medical Center, Boston, MA, USA

* To whom correspondence should be addressed: iam391@mail.harvard.edu

Supplementary Table 1. Comparison of primer and probe design approaches to ensure high coverage. 3

Supplementary Table 2. Database HIV-1 subtype distribution compared to patient population prevalence............. 4

Supplementary Table 3. Frequency of probe-binding site sequence variants around HIV-1 RT codon 103. 5

Supplementary Table 4. Comparison of standard consensus primers to PANDAA primers..................................... 6

Supplementary Table 5. PDR degeneracy at positions with 1–5% nucleotides present. 7

Supplementary Table 6. Effects of PDR degeneracy on PANDAA sensitivity... 8

Supplementary Table 7. Effect of a -6G sequential adaptation primer on other templates. 9

Supplementary Table 8. Individual and total allele-specific pro-amplification primers on PANDAA performance. .. 10

Supplementary Table 9. Probe-binding site alleles relative to wild-type and DRM synthetic templates. 11

Supplementary Table 10. Probe-binding site alleles in patient samples. 12

Supplementary Table 11. Agreement between PANDAA and population sequencing for four DRMs.................... 13

Supplementary Table 12. Diagnostic sensitivity and specificity of PANDAA.. 14

Supplementary Fig. 1. Resolution of a single PCR product by agarose gel electrophoresis................................. 15

Supplementary Fig. 2. Resolution of a single PCR product using a range of PDR degeneracy with SYBR qPCR. 16

Supplementary Fig. 3. Adaptation of the primer-binding region by PANDAA... 17

Supplementary Fig. 4. PANDAA in a one-step RT–qPCR with RNA.. 18

Supplementary Fig. 5. Principle behind Pro-amp (model).. 19

Supplementary Fig. 6. Template Design... 20

Supplementary Table 1. Comparison of primer and probe design approaches to ensure high coverage.

Approach	Methodology	Sequences Used	K65 Probe-Binding Alleles	Advantages	Disadvantages
**Subtype Agnostic Consensus Nucleotide**	1. Calculation of nucleotide frequency at each position.<br>2. Degenerate bases selected >5% frequency.	All available sequences are used to determine the consensus sequence.	ATAAARA-RAARGAY<br>16-fold degenerate	Allows quick determination of nucleotide positions display high diversity.	Bias arises from subtypes over-represented in the sequence database.
**Equally-Weighted Consensus Nucleotide**	3. Calculation of nucleotide frequency at each position.<br>4. Degenerate bases selected >5% frequency.	Uses an equivalent number of sequences from each subtype.	ATAAARA-RAARGAY<br>16-fold degenerate	Normalizes for bias due to over-or under-represented subtypes in the sequence dataset.	Gives undue significance to variability in low prevalent subtypes such as circulating recombinant forms.
**Equally-Weighted Alleles**	5. Selection of equal number of sequences from each subtype.<br>6. Determine unique probe-binding site alleles.<br>7. Calculate frequency for each unique allele.	Uses an equivalent number of sequences from each subtype.	ATAAAGA-AAAAGAC [40.5%]<br>ATAAAGA-AAAGGAC [18.8%]<br>ATAAAGA-AAAAGAT [17.6%]<br>ATAAAAA-GAAGGAC [13.9%]<br>ATAAAGA-AAAGGAT [3.8%]<br>ATAAAAA-GAAAGAC [2.4%]	Normalizes for bias due to over-or under-represented subtypes in the sequence dataset.	Overcalls the prevalence of the most common allele due to it being the most prevalent allele in subtypes D, F, and G, which have low patient prevalence.
**Prevalence-Weighted Alleles**	8. Calculate frequency for each unique allele per subtype.<br>9. Adjust allele frequency based on subtype prevalence.<br>10. Combine weighted allele frequencies.	All available sequences are used to determine unique probe-binding site alleles per subtype.	ATAAAAA-GAAGGAC [44.4%]<br>ATAAAGA-AAAAGAC [21.0%]<br>ATAAAGA-AAAAGAT [11.4%]<br>ATAAAGA-AAAGGAC [9.1%]<br>ATAAAAA-GAAAGAC [6.5%]<br>ATAAAAA-GAAGGAT [2.8%]	Increases the coverage of oligonucleotide matches in patients with unknown subtypes. The most prevalent probe-binding site here is most common in subtype C and non-A-to-G subtypes.	Some bias may occur due to HIV-1C being the most prevalent subtype.

“-” indicates the position of the DRM-discriminating SNV

Supplementary Table 2. Database HIV-1 subtype distribution compared to patient population prevalence.

Subtype	Prevalence	Database Distribution
**A**	9.2%	5.8%
**01_AE**	4.4%	5.8%
**02_AG**	7.3%	2.7%
**B**	10.8%	55.9%
**C**	50.5%	16.8%
**D**	4.4%	3.2%
**F**	0.6%	0.9%
**G**	4.7%	1.0%
**Other**	8.0%	7.9%

From https://www.hiv.lanl.gov/components/sequence/HIV/geo/misc/HIV-1/all/db_world_data.html

Supplementary Table 3. Frequency of probe-binding site sequence variants around HIV-1 RT codon 103.

Allele	Prevalence	Cumulative	Accession	15-nt Probe-Binding Site	Country	Subtype	RT Mutations
**Naturally Occurring Sequence Variants**							
**001**	84.8%	84.8%	AF443074	AAAAGAA A/C AAATCAG	Botswana	C	
**002**	4.2%	88.9%	EF550533	....G....	Ghana	CRF02_AG	
**003**	4.2%	93.2%	HM025038	.G.....	Brazil	B	M41L, L100I
**004**	1.9%	95.0%	AF361877	.... .G.....	Tanzania	C	V90I
**005**	1.9%	96.9%	GQ371960	..C....	USA	B	
**007**	0.8%	97.7%	KC204777	...G...	Botswana	C	
**010**	0.4%	98.1%	EF394236	....T.	China	B	
**011**	0.4%	98.5%	JX447900	.G..A..	Thailand	CRF01_AE	
**012**	0.2%	98.7%	GU345235	....C.	China	B	
**013**	0.2%	98.9%	KC238186	....G.	Australia	B	D67N, T69D, K70R
**014**	0.2%	99.1%	JN000043	.G..... . ..G....	Cuba	CRF18_cpx	

**Co-Expressed DRMs in Probe-Binding Site**							
**006**	-	-	KJ176492	....A	South Africa	C	V106M
**015**	-	-	FJ688264	C......	Cameroon	A	D67E, V75M, K101P
**008**	-	-	KC169338	......G	Mexico	B	K103R
**009**	-	-	KF026125	G......	Ethiopia	C	K101R
**016**	-	-	DQ878845	.C.....	Italy	B	K101H
**017**	-	-	DQ518437	......G . ...A	Bahamas	B	K103R, V106I
**018**	-	-	JN671242	..C.... . .G.....	Argentina	B	K102Q, K104R
**019**	-	-	AM181805	.G..... . ...A	Burkina Faso	CRF06_cpx	V106I

Supplementary Table 4. Comparison of standard consensus primers to PANDAA primers.

A single mismatch was present in both the forward and reverse consensus primers: a C:T mismatch toward the 5' end of the forward primer, and a G:A mismatch at the penultimate 3' position of the reverse primer. Both positions were degenerate bases in the PANDAA primers.

	Forward (5'–3')	Reverse (3'–5')
**Template 001**	TACCACACCCAGCAGGGTTAA	TGACAGTACTGGATGTGGGGGATGCATATT
**Consensus Primers**	.......T.............	.A............................
**PANDAA Primers**	....N..Y..H..R..DY...	.R.....RY.R.....R..D........Y.

Median Cq values (interquartile ranges) of six replicates using either consensus or PANDAA primers across a dynamic range of 001 template DNA.

Copy Number	Consensus	PANDAA
**10^6**	21.2 (IQR, 21.2 to 21.3)	24.7 (IQR, 24.6 to 24.9)
**10^5**	25.6 (IQR, 25.5 to 25.6)	28.2 (IQR, 28.2 to 28.3)
**10^4**	28.9 (IQR, 28.7 to 28.9)	32.0 (IQR, 31.9 to 32.0)
**10^3**	36.0 (IQR, 35.6 to 36.3)	35.6 (IQR, 35.5 to 35.8)
**10^2**	42.5 (IQR, 41.3 to 42.8)	39.3 (IQR, 39.2 to 39.3)

Supplementary Table 5. PDR degeneracy at positions with 1–5% nucleotides present.

	2830F-Forward PANDAA Primer				2896R-Reverse PANDAA Primer	
**Consensus**	**95%**	**96–97%**	**98%**	**99%**	**95%**	**99%**
**Degeneracy**	288	1,536	2,048	18,432	384	1,536

We varied the PDR to represent the 95–99% consensus (i.e. 1–5% nucleotides present).The forward PANDAA primer, 2830F, had four PDR variations based on nucleotide frequency: 95%, 96/97% (no difference in degeneracy), 98%, and 99%. The reverse primer, 2896R, had only two PDR variations: 95% and 99%. The lowest combined degeneracy with a forward and reverse primer was 672-fold with 2830F+95% + 2896R-95%, and the highest degeneracy was 19,968-fold with the 99% consensus variants (18,432-fold + 1,536-fold).

Supplementary Table 6. Effects of PDR degeneracy on PANDAA sensitivity.

Template 001

With the 001 template, regardless of PDR degeneracy in the 2896R PANDAA primer, incorporating nucleotides with a frequency of 2–4% (i.e. 96–98% consensus) into the 2830F PANDAA primer had a modest effect, increasing the sensitivity by ~1.25-fold. Including degenerate bases when a nucleotide was present at 1% (i.e. 99% consensus) reduced sensitivity by ~1.08-fold.

	**2896R–95% PDR Degeneracy**				**2896R–99% PDR Degeneracy**			
**2830F PDR**	**95%**	**96–97%**	**98%**	**99%**	**95%**	**96–97%**	**98%**	**99%**
**Median Cq**	30.02	29.68	29.69	30.12	29.83	29.51	29.48	29.93
**St Dev**	0.09	0.03	0.14	0.10	0.03	0.08	0.10	0.15
**ΔCq**	-	-0.33	-0.33	0.11	-	-0.32	-0.35	0.11
**Fold Change**	-	1.26	1.25	-1.08	-	1.24	1.27	-1.08

Template 014

A greater effect of PDR degeneracy on sensitivity was observed when using template 014, which requires adaptation by both forward and reverse PANDAA primers. Degenerate bases introduced at positions with a nucleotide frequency ≥3% enhanced sensitivity by ~22-fold. A similar trend in increased sensitivity was apparent when using a primer representing the 98% consensus, with a loss in effect occurring when degeneracy matched nucleotides with a frequency of 1%.

	**2896R-95% PDR Degeneracy**				**2896R-99% PDR Degeneracy**			
**2830F PDR**	**95%**	**96–97%**	**98%**	**99%**	**95%**	**96–97%**	**98%**	**99%**
**Median Cq**	34.93	30.98	31.29	34.69	35.02	30.55	31.33	34.91
**St Dev**	0.04	0.10	0.05	0.06	0.45	0.15	0.04	0.03
**ΔCq**	-	-3.96	-3.64	-0.25	-	-4.47	-3.69	-0.10
**Fold Change**	-	**15.54**	12.46	1.19	-	**22.19**	12.92	1.07

Supplementary Table 7. Effect of a -6G sequential adaptation primer on other templates.

Sample Name	dCq	Fold Change	Sequence	Mismatch
**AAA 001**	0.7	-1.5	AAAAGAA C AAATCAG	
**AAA 002**	0.3	-1.2	....G....	+3G
**AAA 003**	-8.6	203.2	.G.....	-6G
**AAA 004**	0.6	-1.5	.... .G.....	+2G
**AAA 005**	0.8	-1.7	..C....	-5C
**AAA 006**	0.1	-1.1	....A	+7A
**AAA 007**	1.1	0.5	...G...	-4G
**AAA 008**	0.9	-1.8	......G	-1G
**AAA 009**	0.8	-1.6	G......	-7G
**AAA 010**	0.6	-1.4	....T.	+6T
**AAA 011**	-2.8	5.5	.G..A..	-6G, -3A
**AAA 012**	0.7	-1.5	....C.	+6C
**AAA 013**	0.9	-1.7	....G.	+6G
**AAA 014**	-0.3	1.2	.G..... . ..G....	-6G, +3G
**AAA 015**	1.4	0.4	C......	-7C
**AAA 016**	5.8	0.0	.C.....	-6C
**AAA 017**	1.0	-1.8	......G . ...A	-1G, +7A
**AAA 018**	0.8	-1.6	..C.... . .G.....	-5C, +2G
**AAA 019**	-2.4	4.3	.G..... . ...A	-6G, +7A
**Median**	0.7	-1.4		
**25th percentile**	0.2	-1.6		
**75th percentile**	0.9	0.5		

Supplementary Table 8. Individual and total allele-specific pro-amplification primers on PANDAA performance.

Template	Probe-Binding Site	Mismatch	Single Pro-Amp dCq	Total Pro-Amp dCq
**Probe**	AAAAGAA C AAATCAG			
**001**	....			
**002**	....G....	+3G	-0.6	0.1
**003**	.G.....	-6G	-8.3	-3.2
**004**	.... .G.....	+2G	-1.2	0.4
**005**	..C....	-5C	0.0	-0.5
**006**	....A	+7A	-1.3	0.1
**007**	...G...	-4G	0.1	0.1
**008**	......G	-1G	0.0	0.2
**009**	G......	-7G	-1.0	-0.7
**010**	....T.	+6T	-0.6	0.0
**011**	.G..A..	-6G, -3A	-7.2	-7.6
**012**	....C.	+6C	1.2	-0.4
**013**	....G.	+6G	0.0	0.1
**014**	.G..... . ..G....	-6G, +3G	-0.4	-0.1
**015**	C......	-7C	-1.1	-0.5
**016**	.C.....	-6C	-2.3	0.2
**017**	......G . ...A	-1G, +7A	-1.0	0.4
**018**	..C.... . .G.....	-5C, +2G	-1.0	0.2
**019**	.G..... . ...A	-6G, +7A	-1.9	-1.3

Pro-Amp: Pro-Amplification

Supplementary Table 9. Probe-binding site alleles relative to wild-type and DRM synthetic templates.

	K65R *			K103N §			Y181C			M184V / I †		
	5' ADR	SNP	3'ADR	5' ADR	SNP	3'ADR	5' ADR	SNP	3'ADR	5' ADR	SNP	3'ADR
Probe	**ATAAAGA**	**A/G**	**RAAAGAC**	**AAAAGAA**	**A/C**	**AAATCAG**	**TTATCT**	**A/G**	**YCAATA**	**TCAATAT**	**A/GTR**	**GATGA**
										**AATAT**	**ATG/A**	**GATGACT**
Allele 001	.....A.		...G...	....		......A	......		......	....		....
Allele 002	....		....	....		..G...A	.C....		......	C		.....T.
Allele 003	....		......T	.G.....		......A	.G....		......	C......		....
Allele 004	....		...G...	....		.G....A	....T.		......	C.....C		.....T.
Allele 005	.....A.		....	..C....		......A	......		...G..	...G...		.....T.
Integrated WT												
001	ATAAAAA	A	RAAGGAC	AAAAGAA	A	AAATCAG	TTATCT	A	TCAATA	TCAATAT	ATG	GATGACT
002	ATAAAGA	A	RAAAGAC	AAAAGAA	A	AAGTCAG	TCATCT	A	TCAATA	TCAATAC	ATG	GATGATT
003	ATAAAGA	A	RAAAGAT	AGAAGAA	A	AAATCAG	TGATCT	A	CCAATA	CCAATAT	ATG	GATGACT
004	ATAAAGA	A	RAAGGAC	AAAAGAA	A	AGATCAG	TTATTT	A	CCAATA	CCAATAC	ATG	GATGATT
005	ATAAAAA	A	RAAAGAC	AACAGAA	A	AAATCAG	TTATCT	A	TCAGTA	TCAGTAT	ATG	GATGATT
Integrated DRM ‡												
001	ATAAAAA	G	RAAGGAC	AAAAGAA	C	AAATCAA	TTATCT	G	TCAATA	TCAATAT	RTA	GATGACT
002	ATAAAGA	G	RAAAGAC	AAAAGAA	C	AAGTCAA	TCATCT	G	TCAATA	TCAATAC	RTA	GATGATT
003	ATAAAGA	G	RAAAGAT	AGAAGAA	C	AAATCAA	TGATCT	G	CCAATA	CCAATAT	RTA	GATGACT
004	ATAAAGA	G	RAAGGAC	AAAAGAA	C	AGATCAA	TTATTT	G	CCAATA	CCAATAC	RTA	GATGATT
005	ATAAAAA	G	RAAAGAC	AACAGAA	C	AAATCAA	TTATCT	G	TCAGTA	TCAGTAT	RTA	GATGATT

* K65 probes were designed against the second most prevalent allele (002) to minimize the number of single-nucleotide variants to be adapted in the probe-binding site.

§ K103 probe have a G>A mismatch at the 3' terminus as the Integrated DRM templates also incorporated the V106M mutation.

† Probes for 184V and 184I differ in the positioning of the discriminating SNP. For 184V, an A → G substitution occurs at the 1st nucleotide of codon 184, and for 184I a G → A substitution at the third nucleotide. This leads to a 2-nt downstream shift in the probe-binding site for 184I. As both DRMs are adapted and amplified by the same PANDAA primers the 5' and 3' ADRs must cover the cumulative probe-binding region.

‡ The M184V mutation can represent a change from ATG → GTG or GTA; the M184I mutation is ATG → ATA. To incorporate both 184V and 184I mutations into a single synthetic template, the 184 codon is given as RTA (GTA and ATA) in the Integrated DRM templates.

Supplementary Table 10. Probe-binding site alleles in patient samples.

K65	Count	
ATA AAA A-G AAG GAC	47	65.3%
ATA AAA A-G AAA GAC	12	16.7%
ATA AAG A-A AAA GAC	8	11.1%
ATA AAA A-G AAG GAT	3	4.2%
ATT AAA A-G AAG GAC	1	1.4%
ATA AAA A-G AAG GGC	1	1.4%

K103	Count	
AA AAG AA- AAA TCA G	57	79.2%
AA AAG AA- AAA TCA A	2	2.8%
AA AAG AA- AGA TCA G	2	2.8%
AG AAG AA- AAA TCA G	4	5.6%
AA AAG AA- AAG TCA G	2	2.8%
AA CAG AA- AAA TCA G	3	4.2%
AA AAG AA- AAG TCA A	1	1.4%
AA AAG AA- AAA TCA R	1	1.4%

Y181	Count	
GTC ATC T-T CAA TAT	41	56.9%
GTT ATC T-T CAA TAC	13	18.1%
GTT ATC T-T CAA TAT	8	11.1%
GTT ATC T-C CAA TAT	4	5.6%
GTC ATC T-T CAG TAT	4	5.6%
GTC ATC T-T CAA TAC	1	1.4%
RTT ATC T-C CAA TAT	1	1.4%

M184I	Count	
AAT --- TGG ATG ACT	49	68.1%
AAT --- TGG ATG ATT	14	19.4%
AAT --- TAG ATG ACC	3	4.2%
AGT --- TGG ATG ACT	3	4.2%
AAT --- TGG ATG ACC	1	1.4%
AGT --- TGG ATG ATT	1	1.4%
AAT --- TAG ATG ACT	1	1.4%

M184V	Count	
TC AAT --- TGG ATG A	59	81.9%
CC AAT --- TGG ATG A	5	6.9%
TC AAT --- TAG ATG A	4	5.6%
TC AGT --- TGG ATG A	4	5.6%

Supplementary Table 11. Agreement between PANDAA and population sequencing for four DRMs.

NRTI DRMs

K65R

	DRM [Sanger]	WT [Sanger]
DRM [PANDAA]	18	3
WT [PANDAA]	0	51

		95% CI
Sensitivity	100.0%	81.5% - 100.0%
Specificity	94.4%	84.6% - 98.8%
Accuracy	95.8%	

M184VI

	DRM [Sanger]	WT [Sanger]
DRM [PANDAA]	15	0
WT [PANDAA]	0	57

		95% CI
Sensitivity	100.0%	78.2% - 100.0%
Specificity	100.0%	93.7% - 100.0%
Accuracy	100.0%	

NNRTI DRMs

K103N

DRM [PANDAA]	11	4
WT [PANDAA]	0	57

		95% CI
Sensitivity	100.0%	71.5% - 100.0%
Specificity	93.4%	84.1% - 98.2%
Accuracy	94.4%	

Y181C

	DRM [Sanger]	WT [Sanger]
DRM [PANDAA]	24	0
WT [PANDAA]	0	48

		95% CI
Sensitivity	100.0%	85.8% - 100.0%
Specificity	100.0%	92.6% - 100.0%
Accuracy	100.0%	

Supplementary Table 12. Diagnostic sensitivity and specificity of PANDAA

First-Line ART Failure

DRM [PANDAA]	31	1
WT [PANDAA]	1	39

		95% CI
Sensitivity	96.9%	83.8% - 99.9%
Specificity	97.5%	86.8% - 99.9%
Accuracy	97.2%	

NRTI Drug Class Failure

	DRM [Sanger]	WT [Sanger]
DRM [PANDAA]	23	3
WT [PANDAA]	0	46

		95% CI
Sensitivity	100.0%	85.2% - 100.0%
Specificity	93.9%	83.1% - 98.7%
Accuracy	95.8%	

NNRTI Drug Class Failure

	DRM [Sanger]	WT [Sanger]
DRM [PANDAA]	28	0
WT [PANDAA]	4	40

		95% CI
Sensitivity	87.5%	71.0% - 96.5%
Specificity	100.0%	91.2% - 100.0%
Accuracy	94.4%	

Supplementary Fig. 1. Resolution of a single PCR product by agarose gel electrophoresis.

To confirm that amplification efficiency was not proportionally inhibited as the number of overlapping nucleotides increased, PANDAA reactions utilizing 13–17-nt probes were resolved on a 4% agarose gel, demonstrating comparable band intensities of a 66-bp amplicon across all probe lengths at both 10^3 and 10^4 DNA copies per reaction (middle lane: 10-bp DNA ladder). Furthermore, no non-specific products were evident. Note: this is the full, uncropped gel image that shown in Figure 2d.

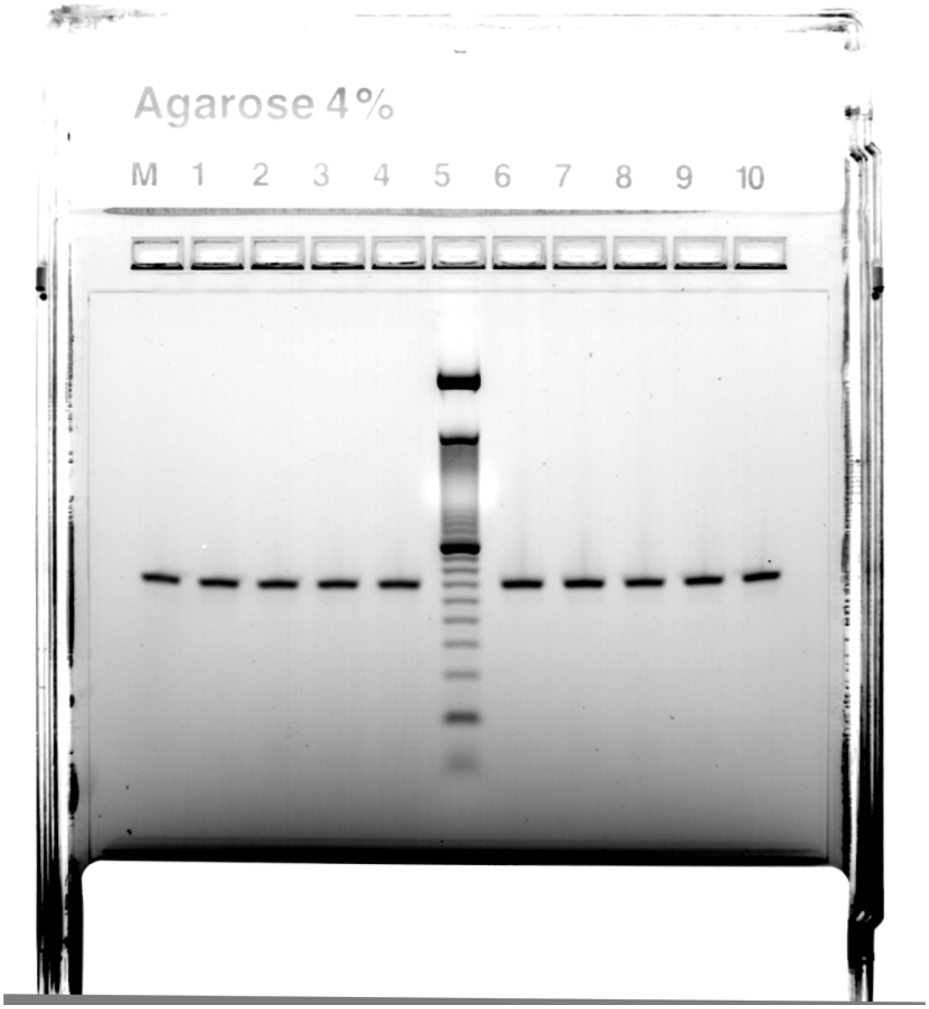

Supplementary Fig. 2. Resolution by SYBR qPCR of a single PCR product using a range of PDR degeneracy.

Each 2830F PDR variant was used with either the 2896R-95% or 2896R-99% in a SYBR green qPCR with the 014 template. A similar pattern of increased reaction efficiency to that with probe-based PANDAA was observed: an increase in degeneracy up to 96–98% consensus improved amplification by both SYBR and probe-based qPCR compared to 95% consensus, then subsequently reduced amplification. This suggests that there is a tipping point after which an increase in PDR degeneracy reduces amplification efficiency. This effect was noticeable only with the forward primer and not the reverse primer because 2830F-99% has a 18,432-fold degeneracy whereas 2896R-99% has only a 1,536-fold degeneracy. The lower amplicon Tm and broader melt curve when using the 2896R-99% primer is indicative of the wider range of GC% content compared to 2896R-95% (25.8 - 58.1% vs. 29.0–54.8%), and therefore the wider range of primer Tm (63.0–78.7°C vs. 64.2–77.3°C).

SYBR qPCR with 2896R-95% Reverse Primer

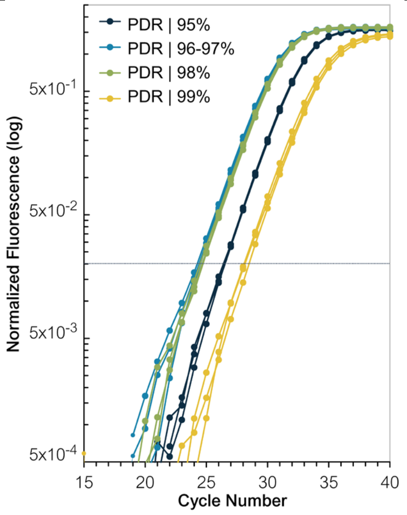

2830F PDR	95%	96–97%	98%	99%
Median Cq	26.31	24.13	24.47	28.14
St Dev	0.01	0.14	0.07	0.16
ΔCq	-	-2.17	-1.84	1.83
Fold Change	-	4.51	3.58	-3.55
Amplicon T_m	73.3°C	72.8°C	73.0°C	72.2°C

Melt Curve

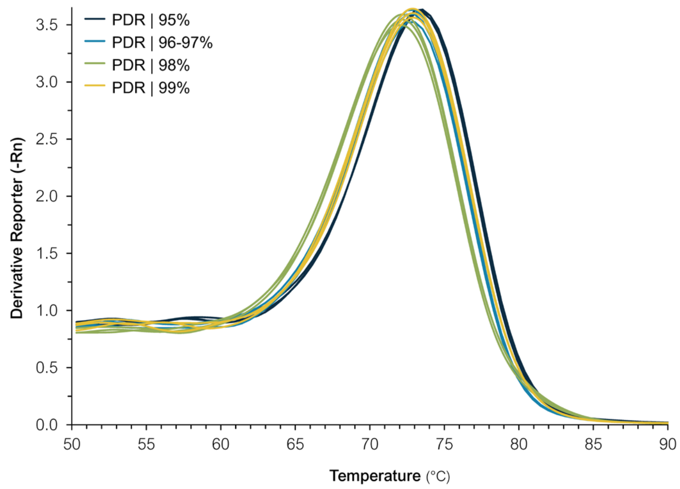

SYBR qPCR with 2896R-99% Reverse Primer

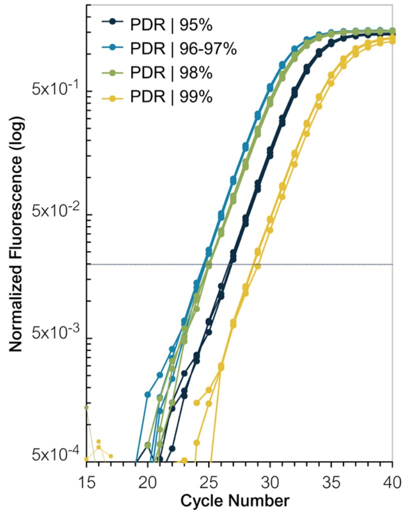

2830F PDR	95%	96–97%	98%	99%
Median Cq	26.69	24.62	24.98	28.79
St Dev	0.10	0.08	0.15	0.23
ΔCq	-	-1.68	-1.32	2.49
Fold Change	-	3.21	2.50	-5.60
Amplicon T_m	72.0°C	71.4°C	71.4°C	71.4°C

Melt Curve

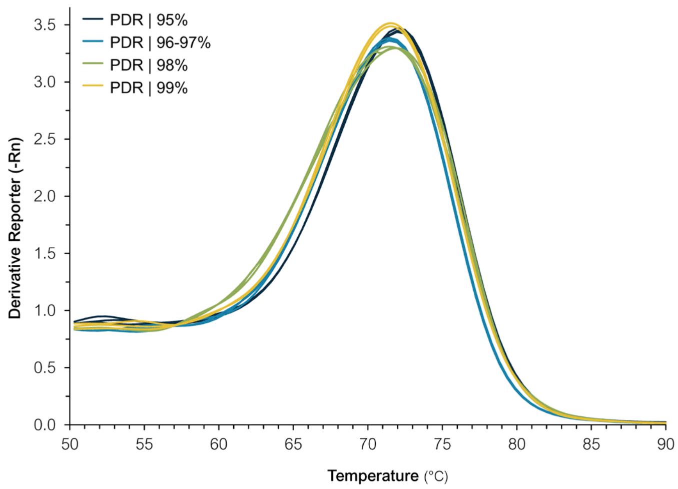

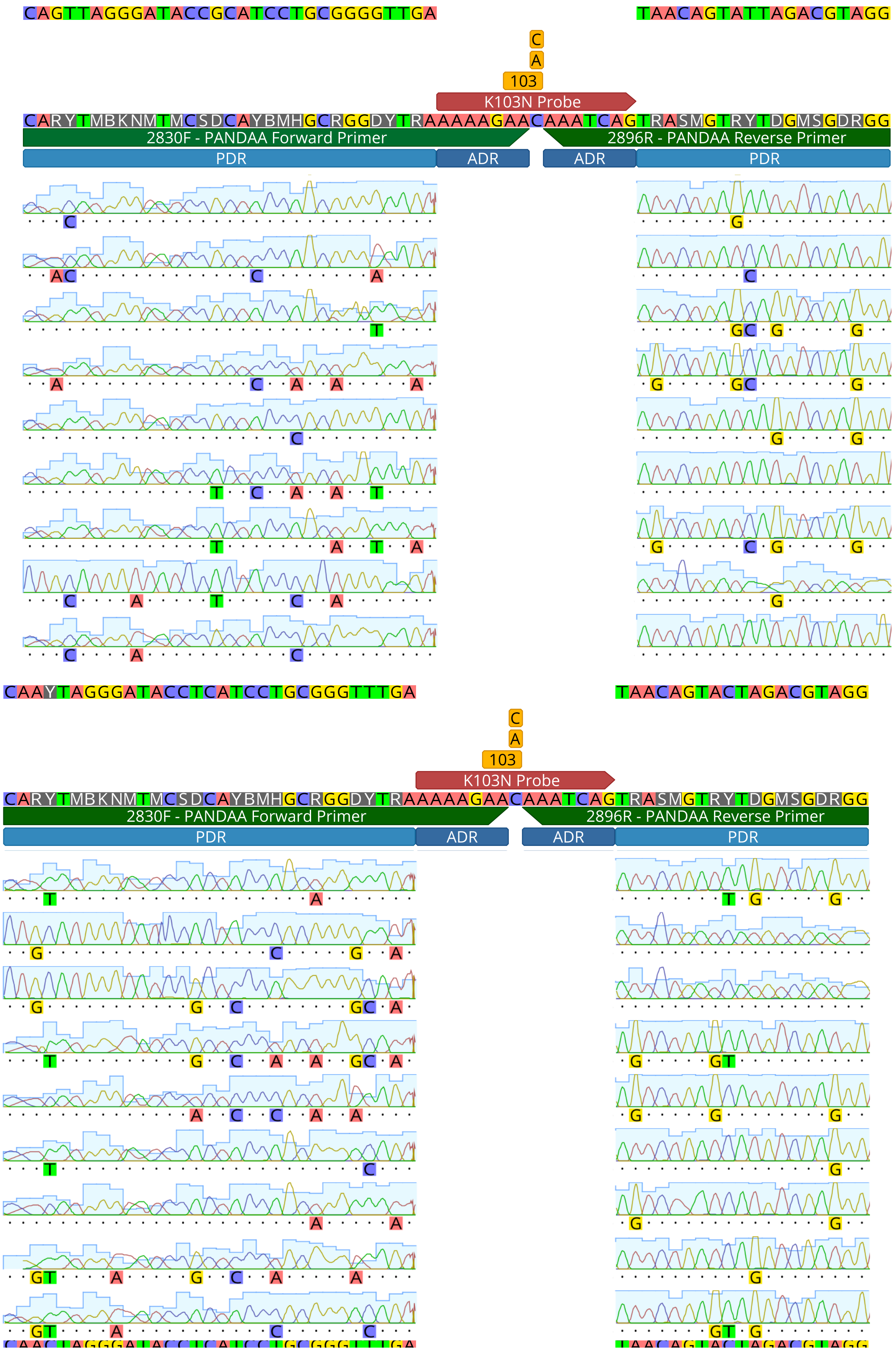

Supplementary Fig. 3. Adaptation of the primer-binding region by PANDAA.

66-bp amplicons, which were derived from PANDAA performed on synthetic DNA templates containing the 001 probe-binding site allele, were cloned into the PCR Cloning Kit (New England Biolabs; Ipswich, MA, USA), and individual clones were sequenced using the associated cloning analysis forward and reverse primers. Representative chromatograms from two DNA templates with different primer-binding sites are shown.

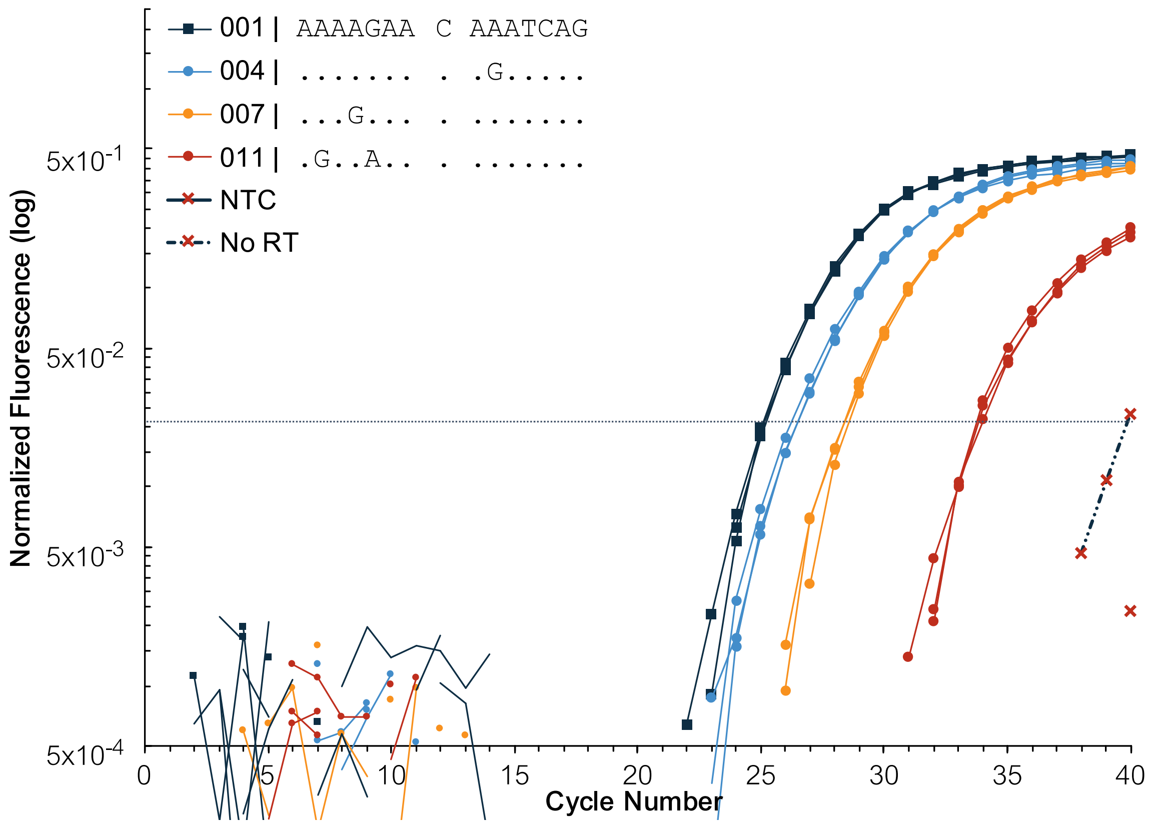

Supplementary Fig. 4. PANDAA in a one-step RT–qPCR with RNA.

Synthetic RNA was derived from synthetic DNA constructs using the HiScribe T7 High Yield RNA Synthesis Kit, NEB. After in vitro transcription, DNA was removed using RQ1 RNase-Free DNase and RNA was purified using RNeasy MiniElute Cleanup Kit (Qiagen; Hilden, Germany) with additional on-column DNA digestion. Single-stranded RNA was quantified using the Qubit RNA High Sensitivity assay (Life Technologies; Carlsbad, CA, USA) and diluted in dH_2O supplemented with S. cerevisiae carrier tRNA at 50 ng/µL (Sigma Aldrich).

10^5 copies of RNA derived from templates 001, 004, 007, and 011 were used in a PANDAA reaction with 15U MMLV RT (NEB) added to Kapa Probe Fast Master Mix in a final volume of 10µL. In a one-step RT–qPCR, the reaction was incubated at 42°C for 15 minutes followed by the standard cycling conditions for the K103 PANDAA. dH_2O with carrier RNA was included in a no RT control to verify no carryover DNA contamination from the RNA synthesis. Templates 004 and 007, which have a probe-binding site mismatch in the 3' and 5' adaptor regions, respectively, demonstrated similar sensitivity in a one-step RT–qPCR. Template 011, which has two mismatches in the 5' adaptor region, was amplified less efficiently, which was also demonstrated using DNA.

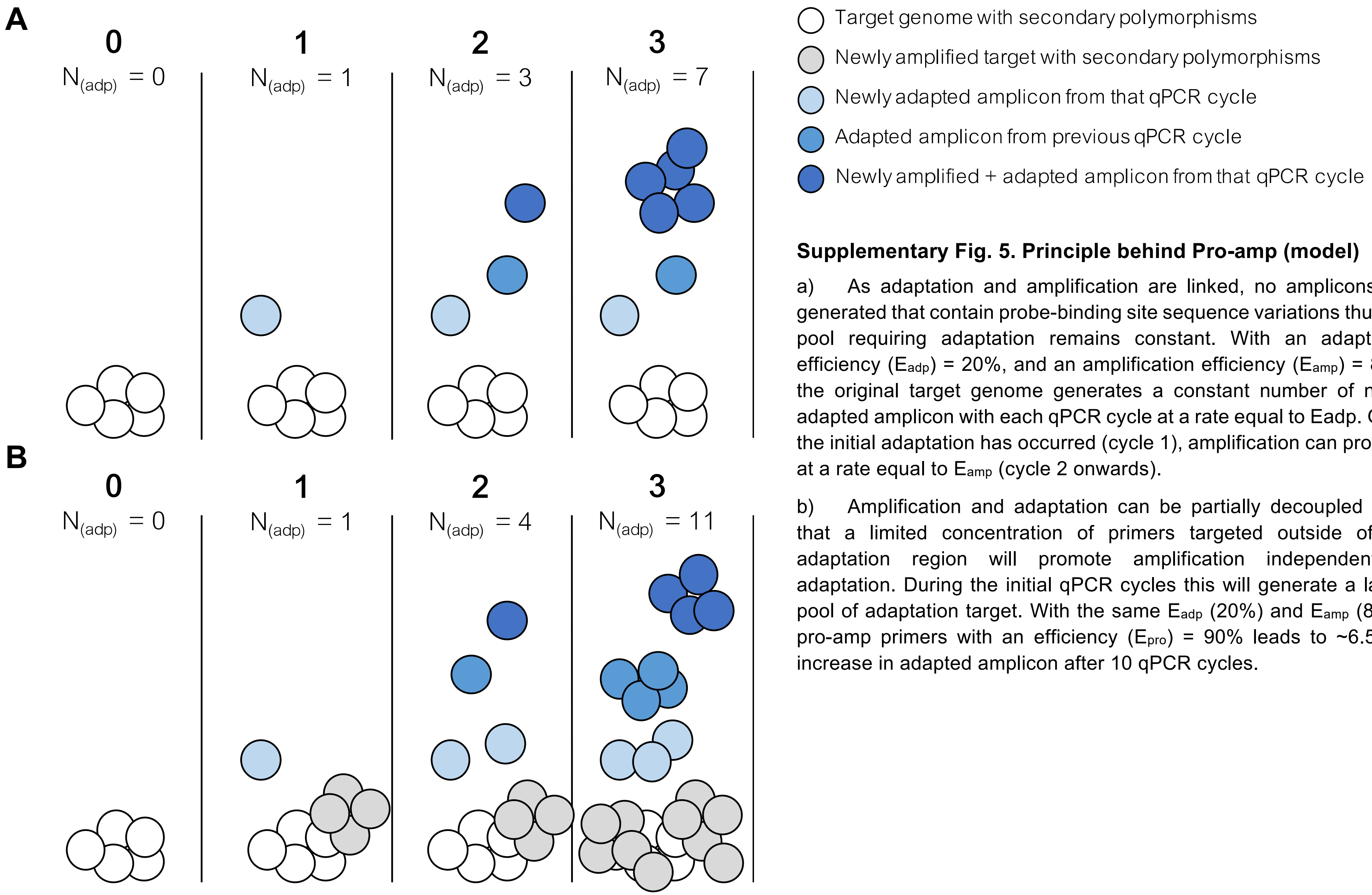

Supplementary Fig. 5. Principle behind Pro-amp (model)

a) As adaptation and amplification are linked, no amplicons are generated that contain probe-binding site sequence variations thus the pool requiring adaptation remains constant. With an adaptation efficiency (E_{adp}) = 20%, and an amplification efficiency (E_{amp}) = 85%, the original target genome generates a constant number of newly adapted amplicon with each qPCR cycle at a rate equal to Eadp. Once the initial adaptation has occurred (cycle 1), amplification can proceed at a rate equal to E_{amp} (cycle 2 onwards).

b) Amplification and adaptation can be partially decoupled such that a limited concentration of primers targeted outside of the adaptation region will promote amplification independent of adaptation. During the initial qPCR cycles this will generate a larger pool of adaptation target. With the same E_{adp} (20%) and E_{amp} (85%), pro-amp primers with an efficiency (E_{pro}) = 90% leads to ~6.5-fold increase in adapted amplicon after 10 qPCR cycles.

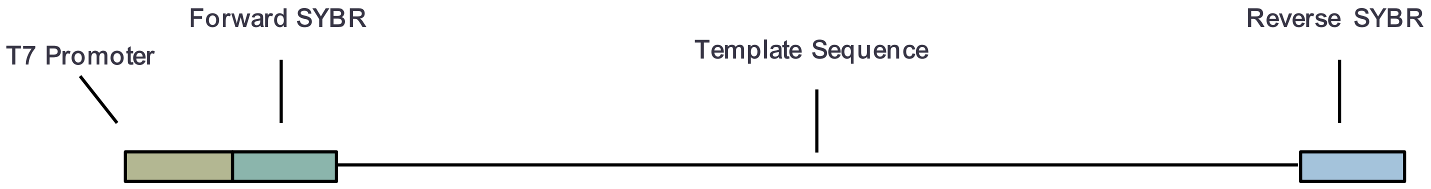

SYBR primers for quantification:

```
Fwd 5'-GCT CCT CTG GAA AGG TGA AG
Rev 5'-GCG GAT AAC AAT TTC ACA CAG G
```

Supplementary Fig. 6. Template Design.

Synthetic double-stranded DNA was designed to evaluate the sensitivity and specificity of PANDAA. The 5' region of the template contains the promoter for T7 RNA polymerase to derive synthetic single-stranded RNA. Immediately downstream of the T7 promoter, and at the 3' terminus, we included optimized primer-binding sites to allow SYBR green confirmation of the template copy number. Lyophilized geneStrings (Life Technologies) were resuspended in TE buffer to obtain a template master stock at 10^{10} copies/µL. Templates were subsequently diluted in dH_2O supplemented with carrier tRNA from Saccharomyces cerevisiae at 0.05 µg/µL (Sigma Aldrich) to provide a dilution series from 10^6 copies/µL to 100 copies/µL.
