# Supplementary material for: PANDAA intentionally violates conventional qPCR design to enable durable, mismatch-agnostic detection of highly polymorphic pathogens
Source: Commun Biol. 2021 Feb 18;4:227. doi: 10.1038/s42003-021-01751-9 (PMC7892852; doi:10.1038/s42003-021-01751-9)
Supplement: Supplementary file 3 — Description of Additional Supplementary Files [file 42003_2021_1751_MOESM3_ESM.pdf]

Description of Additional Supplementary Files

File name: Supplementary Data 1

Description: Source data for all graphs.
