# Supplementary material for: PANDAA intentionally violates conventional qPCR design to enable durable, mismatch-agnostic detection of highly polymorphic pathogens
Source: Commun Biol. 2021 Feb 18;4:227. doi: 10.1038/s42003-021-01751-9 (PMC7892852; doi:10.1038/s42003-021-01751-9)
Supplement: Supplementary file 5 — Reporting Summary [file 42003_2021_1751_MOESM5_ESM.pdf]

nature research

Corresponding author(s): Iain J. MacLeod

Last updated by author(s): Nov 15, 2020

Reporting Summary

Nature Research wishes to improve the reproducibility of the work that we publish. This form provides structure for consistency and transparency in reporting. For further information on Nature Research policies, see our Editorial Policies and the Editorial Policy Checklist.

Statistics

For all statistical analyses, confirm that the following items are present in the figure legend, table legend, main text, or Methods section.

n/a	Confirmed	
☐	☒	The exact sample size (*n*) for each experimental group/condition, given as a discrete number and unit of measurement
☒	☐	A statement on whether measurements were taken from distinct samples or whether the same sample was measured repeatedly
☐	☒	The statistical test(s) used AND whether they are one- or two-sided<br>*Only common tests should be described solely by name; describe more complex techniques in the Methods section.*
☒	☐	A description of all covariates tested
☒	☐	A description of any assumptions or corrections, such as tests of normality and adjustment for multiple comparisons
☐	☒	A full description of the statistical parameters including central tendency (e.g. means) or other basic estimates (e.g. regression coefficient) AND variation (e.g. standard deviation) or associated estimates of uncertainty (e.g. confidence intervals)
☒	☐	For null hypothesis testing, the test statistic (e.g. *F*, *t*, *r*) with confidence intervals, effect sizes, degrees of freedom and *P* value noted<br>*Give P values as exact values whenever suitable.*
☒	☐	For Bayesian analysis, information on the choice of priors and Markov chain Monte Carlo settings
☒	☐	For hierarchical and complex designs, identification of the appropriate level for tests and full reporting of outcomes
☐	☒	Estimates of effect sizes (e.g. Cohen's *d*, Pearson's *r*), indicating how they were calculated

Our web collection on statistics for biologists contains articles on many of the points above.

Software and code

Policy information about availability of computer code

Data collection	Data were collected using an ABI 7900 (Applied Biosystems) with software SDS v2.4.
Data analysis	Data were exported from the SDS v2.4 software and analyzed in Microsoft Excel v16.0 and GraphPad Prism v6 for Windows.

For manuscripts utilizing custom algorithms or software that are central to the research but not yet described in published literature, software must be made available to editors and reviewers. We strongly encourage code deposition in a community repository (e.g. GitHub). See the Nature Research guidelines for submitting code & software for further information.

Data

Policy information about availability of data

All manuscripts must include a data availability statement. This statement should provide the following information, where applicable:

- Accession codes, unique identifiers, or web links for publicly available datasets
- A list of figures that have associated raw data
- A description of any restrictions on data availability

The authors declare that the data supporting the findings of this study are available within the paper and its supplementary information files. The PANDAA primer and probe design data are available from the corresponding author [IJM], however, restrictions may apply to the availability of these data due to the proprietary nature of the data and so are not publicly available. PANDAA reagents (combined primers and probes) may be available through a material transfer agreement with the President and Fellows of Harvard College, or Aldatu Biosciences, Inc, which can be facilitated by contacting the corresponding author.

Field-specific reporting

Please select the one below that is the best fit for your research. If you are not sure, read the appropriate sections before making your selection.

- [x] Life sciences
- [ ] Behavioural & social sciences
- [ ] Ecological, evolutionary & environmental sciences

For a reference copy of the document with all sections, see nature.com/documents/nr-reporting-summary-flat.pdf

Life sciences study design

All studies must disclose on these points even when the disclosure is negative.

Sample size

Sequence Sample Size for PANDAA Design:
We searched the Los Alamos HIV public database (http://www.hiv.lanl.gov) for sequences within the genomic region 2550→3501 (HXB2 coordinates) from all subtypes – including recombinants – with a minimum fragment length of 500 nt. We selected a single sequence per patient, resulting in 93,611 sequences at the time of this study.

Sample Size for Single Amplicon Cloning and Sequencing:
A minimum of 45 single amplicon clones were sequenced. By sequencing a minimum of 45 clones, we had 95% confidence that we would detect sequence variants present in the amplicon population with a frequency of ≥10%: with n single amplicons sequenced, the probability (P) of missing a variant after screening n genomes is calculated as f=1-(1-P^(1/n)) when the variant comprises a fraction f (or less) of the virus population. Ref: Rossenkhan, R. et al. tat Exon 1 Exhibits Functional Diversity during HIV-1 Subtype C Primary Infection. Journal of Virology 87, 5732–5745 (2013).

Sample Size for Sequencing of Clinical Samples:
PANDAA using 72 clinical samples from patients with virological failure on NNRTI-based ART, which was defined as HIV RNA ≥400 copies/ml after six months of study enrollment.

Statistics
The agreement between genotyping methods was determined with Pearson's correlation coefficient. Kappa is a measure of the degree of non-random agreement between observers or measurements of the same categorical variable. The agreement was considered as good at kappa 0.60–0.80 and very good at kappa >0.80.

Data exclusions

No data were excluded from the analyses.

Replication

Technical replicate number depended on the final copies/reaction: ≥10^4 (n=4); ≥10^3 (n=8); and <10^3 (n=12). Human genomic DNA at 0.05 ng/reaction (Promega) was included as the non-target nucleic acid negative control (n=8 replicates). All attempts at replication were successful.

Randomization

Clinical samples for resistance genotyping were not randomized; residual plasma samples and PCR amplicons were used from all patients with defined virological failure.

Blinding

Blinding of clinical samples was used in the initial clinical resistance genotyping by PANDAA. The resistance profiles (i.e. which combination of six drug resistance mutations) was unknown to the operator. Confirmatory resistance genotyping by PANDAA was performed unblinded.

Reporting for specific materials, systems and methods

We require information from authors about some types of materials, experimental systems and methods used in many studies. Here, indicate whether each material, system or method listed is relevant to your study. If you are not sure if a list item applies to your research, read the appropriate section before selecting a response.

Materials & experimental systems

n/a	Involved in the study	
x		Antibodies
x		Eukaryotic cell lines
x		Palaeontology and archaeology
x		Animals and other organisms
	x	Human research participants
x		Clinical data
x		Dual use research of concern

Methods

n/a	Involved in the study	
x		ChIP-seq
x		Flow cytometry
x		MRI-based neuroimaging

Human research participants

Policy information about studies involving human research participants

Population characteristics	This study used de-identified PCR amplicon from the Bomolemo study, an observational cohort designed to demonstrate the tolerability and virological response to a fixed-dose efavirenz/tenofovir/emtricitabine ART regimen. This study was conducted in Gaborone, Botswana, between November 2008 and July 2011 by the Botswana-Harvard AIDS Institute and the Botswana Ministry of Health from whom Institutional Review Board approval was received. Reference: Ryan, K. et al. High Rates of Occult Hepatitis B Virus Infection in HIV-Positive Individuals Initiating Antiretroviral Therapy in Botswana. Open Forum Infectious Diseases 4, ofx195 (2017).<br><br>[The above text is already included in the manuscript Materials and Methods section].
Recruitment	N/A
Ethics oversight	Botswana Ministry of Health Institutional Review Board

Note that full information on the approval of the study protocol must also be provided in the manuscript.
